# Simultaneous Pancreas–Kidney Transplantation versus Kidney Alone: Interpreting Neutral Survival and Persistent Metabolic Advantages

**DOI:** 10.3389/ti.2025.15708

**Published:** 2025-12-30

**Authors:** Lorenzo Piemonti

**Affiliations:** 1 Unit of Regenerative Medicine and Organ Transplants, IRCCS Ospedale San Raffaele, Milan, Italy; 2 Università Vita-Salute San Raffaele, Milan, Italy

**Keywords:** kidney transplantation, simultaneous pancreas–kidney transplantation, pancreas transplantation, long-term outcomes, real-world data

For patients with diabetes and end-stage kidney disease, transplantation is the most effective therapy to restore renal function and improve long-term outcomes. The choice between kidney transplant alone and simultaneous pancreas–kidney transplantation has, however, remained complex. The combined procedure offers the possibility of eliminating insulin dependence and achieving stable glycaemic control, while kidney transplant alone represents a technically simpler operation with a well-established safety profile. For many years, reports from individual centres suggested that simultaneous pancreas–kidney transplantation might also provide a survival advantage, reinforcing the idea that it was not only metabolically superior but also prognostically preferable.

Recent analyses from two large datasets invite us to reconsider this narrative. One, based on the global TriNetX real-world network, and the other derived from the U.S. Scientific Registry of Transplant Recipients, both compared outcomes between simultaneous pancreas–kidney and kidney-alone recipients using modern propensity-based methods. Despite differences in design and endpoints, their conclusions align: when baseline characteristics are properly balanced, the survival benefit of simultaneous transplantation is not evident, although the metabolic advantage remains clear and the risk of early complications is somewhat greater. It is worth noting, however, that a survival signal appears to re-emerge within more selected subgroups—particularly among recipients with type 1 diabetes and a leaner phenotype.

The TriNetX analysis included adults aged 18–59 years who received deceased-donor grafts, excluding living donor and multi-organ recipients. One-to-one propensity score matching was used to create comparable cohorts. Outcomes were examined over short- and long-term horizons, ranging from the first post-transplant year to five and ten years. TriNetX’s strength was its breadth of endpoints, which extended beyond survival to include major adverse kidney events, cardiovascular complications, infections, malignancies, and, crucially, glycated haemoglobin. The SRTR analysis, in contrast, drew on the completeness of a national registry, applying overlap propensity score weighting to balance populations. Outcomes focused on patient and graft survival at five and ten years, together with acute rejection and hospital readmission within the first year. Although narrower in scope, SRTR provides a highly reliable picture of transplant-specific endpoints.

Despite these methodological contrasts, both studies delivered consistent findings [1, 2]. Neither identified a survival difference between the two strategies once adjustment was applied. In SRTR, patient survival at five and ten years was almost identical between groups, and TriNetX confirmed this neutrality. Kidney graft survival followed the same pattern. An important nuance is that, within the subset of recipients with type 1 diabetes and a leaner phenotype, SRTR did identify a statistically significant survival advantage for simultaneous transplantation, whereas TriNetX showed a trend in the same direction but without reaching statistical significance. Both studies also highlighted a higher frequency of early complications among simultaneous recipients. In the registry analysis, treated acute rejection during the first year was nearly tripled and hospital readmissions doubled, while the real-world analysis showed a similar though slightly attenuated pattern after matching. In terms of composite renal outcomes, TriNetX suggested a modest early reduction in adverse kidney events for simultaneous recipients, but this advantage was not sustained at later time points. Cardiovascular outcomes were largely neutral. What clearly distinguished the simultaneous group in TriNetX was better metabolic control, with lower glycated haemoglobin consistently observed even after matching. This confirms what is biologically expected: the presence of a functioning pancreas graft translates into restored normoglycemia. To facilitate a more granular comparison across methods and endpoints, the key results of the two studies are summarized in Table 1.

**TABLE 1:** Comparison of SRTR and TriNetX studies on SPKT vs. KTA.

Comparison item	TriNetX analysis	SRTR registry study
Data source	Global, multicenter real-world EHR database	U.S. national transplant registry (2014–2023)
Population	Adults 18–59 with diabetes and ESRD; deceased-donor only; multi-organ excluded	Adults 18–59 with diabetes and ESRD; deceased-donor only; multi-organ excluded
Balancing method	1:1 propensity score matching (caliper 0.1)	Overlap propensity score weighting
Outcomes analysed	Patient survival, kidney graft survival, MAKE, cardiovascular events, infections, malignancies, metabolic outcomes (HbA1c), early complications	Patient survival, kidney graft survival (primary endpoints); 1-year acute rejection and hospital readmission (secondary endpoints)
Time windows	Analyses from day 10 (1-year outcomes) and day 90 (5- and 10-year outcomes)	Kaplan–Meier/Cox at 5 and 10 years; 1-year analyses for rejection and readmission
Patient survival	Pre-matching: SPKT appeared superior. Post-matching: Neutral. Sensitivity: Neutral also in T1D-only and non-obese subgroups	Pre-matching: SPKT appeared superior. Post-matching: Neutral overall. Sensitivity: Survival advantage for SPKT in T1D + lean phenotype subgroup
Kidney graft survival	Pre-matching: apparent advantage for SPKT Post-matching: neutral	Pre-matching: Apparent advantage for SPKT Post-matching: Neutral
Acute rejection (1y)	Pre-matching: higher in SPKT. Post-matching: neutral	Higher in SPKT (owOR ∼2.8)
Hospital readmission (1y)	Pre-matching: Higher in SPKT. Post-matching: Slightly higher in SPKT.	Higher in SPKT (owOR ∼2.0)
Other complications	Early reduction in MAKE; long-term MAKE neutral; cardiovascular endpoints neutral	Not assessed
Metabolic outcomes	HbA1c consistently lower in SPKT (superior glycaemic control)	Not available
Overall conclusion	No survival advantage overall after adjustment; early morbidity higher; clear metabolic superiority	No survival advantage overall after adjustment; higher early rejection and readmission; survival benefit in specific subgroups (T1D + lean phenotype)

The disappearance of the survival advantage often reported in earlier work can be explained by several factors. Historical analyses were strongly influenced by selection bias, as patients chosen for simultaneous transplantation were frequently younger and healthier than those who underwent kidney transplant alone. Once like is compared with like, the curves converge. In addition, the combined procedure carries higher short-term risks linked to its surgical complexity and immunologic challenges, offsetting some of the long-term benefits. Outcomes after kidney transplant alone have also improved over time, narrowing gaps that might previously have been more evident. Finally, it is possible that the vascular and metabolic protection conferred by pancreas transplantation requires a longer time horizon than that captured in current datasets. Many patients remain at substantial risk of death from competing causes, which may obscure benefits that emerge only after decades.

At the same time, both studies must be interpreted with caution. Neither can be considered conclusive, and each has important limitations. Registry analyses such as SRTR excel in completeness but cannot account for metabolic or quality-of-life outcomes, which are highly relevant in this population. Real-world networks like TriNetX provide broader clinical detail but are vulnerable to coding variability and incomplete follow-up. Propensity methods reduce but cannot eliminate residual confounding. Moreover, both analyses exclude living donor transplantation, which in practice remains an important comparator, and neither captures patient-reported outcomes such as hypoglycaemia burden, psychological wellbeing, or daily functioning. A further consideration is that SPKT and KTA recipients are not always fully interchangeable, meaning that even extensive adjustment may not completely resolve baseline differences. Earlier single-centre series—conducted in periods with different standards of diabetes management—consistently reported a survival advantage for SPKT, and the remarkable effort made over decades to refine surgical techniques, perioperative care, and immunosuppression has transformed pancreas transplantation into an increasingly safe and effective procedure. In parallel, contemporary improvements in exogenous insulin therapy and kidney-alone outcomes may have contributed to narrowing the observable survival gap in recent datasets. Subgroup analyses still suggest a possible survival signal in specific phenotypes, such as recipients with type 1 diabetes and lower BMI, although the magnitude and statistical robustness of this effect vary across sources. Together, these considerations highlight that current findings should be viewed as important contributions to an evolving evidence landscape rather than definitive conclusions.

Taken together, these findings should not be viewed as discouraging simultaneous transplantation but rather as refining our understanding of its value. While simultaneous pancreas–kidney transplantation may not consistently demonstrate a survival advantage over kidney-alone transplantation in contemporary adjusted analyses, it continues to provide superior metabolic control and the possibility of insulin independence—outcomes that remain highly meaningful for many patients. For a young adult with type 1 diabetes, the restoration of stable, physiological glycaemic regulation may justify the higher early risks, particularly in the context of a procedure that has become steadily safer and more effective through sustained surgical and perioperative improvements. In addition, emerging subgroup analyses indicate that a survival benefit may persist in specific phenotypes—most notably in recipients with type 1 diabetes and a leaner metabolic profile—suggesting that the value of SPKT is not uniform across all patient categories. For others—especially those with greater comorbidity, different metabolic profiles, or access to living donation—kidney transplant alone may represent the more appropriate strategy. Ultimately, counselling must be individualized, integrating the survival neutrality observed in overall populations with dimensions that registries cannot fully capture, including quality of life, hypoglycaemia burden, and the broader impact of insulin freedom on daily living.

Importantly, these results should in no way diminish the commitment to performing pancreas transplantation or its role within multidisciplinary care. On the contrary, they highlight the need to further strengthen timely referral to high-volume, specialised centres capable of accurately assessing appropriateness and delivering the procedure with the highest standards of safety and expertise. In current practice, a substantial proportion of individuals who could benefit—not only in terms of metabolic restoration but also, for selected subpopulations, in terms of survival—are neither identified nor referred, resulting in missed opportunities for clinically meaningful improvement. Ensuring that eligible patients are correctly evaluated and managed therefore remains an essential priority for the field.

For the field at large, these studies highlight the need for more evidence rather than less. Registries must evolve to incorporate metabolic and patient-centred endpoints, and real-world datasets require further validation and harmonization. International collaborations that combine the completeness of registry data with the granularity of electronic health records could provide a more comprehensive picture of outcomes. Only through such integrated approaches will it be possible to determine whether the metabolic advantage of simultaneous transplantation ultimately translates into reduced vascular complications, preserved organ function, and better long-term health. This need for robust and refined evidence is even more pressing given that a randomized trial comparing SPKT and KTA is neither feasible nor ethically justifiable in this context, making high-quality observational data the only realistic path forward.

The debate between simultaneous pancreas–kidney transplantation and kidney transplant alone is therefore not settled but reframed. The survival benefits once attributed to the combined procedure is less clear under modern analytic methods, yet its metabolic superiority remains unquestionable. More and better evidence is required to fully understand how these dimensions balance over the decades of life after transplantation. Until then, the value of simultaneous transplantation should be appreciated not only through survival curves but also through its potential to transform the daily lives of carefully selected patients.
